# Development of eating skills in infants and toddlers from a neuropediatric perspective

**DOI:** 10.1186/s13052-024-01683-0

**Published:** 2024-06-03

**Authors:** Hermann Kalhoff, Mathilde Kersting, Kathrin Sinningen, Thomas Lücke

**Affiliations:** 1 Research Department of Child Nutrition, University Hospital of Pediatrics and Adolescent Medicine, St. Josef-Hospital, Ruhr-University Bochum, Bochum, Germany; 2 Pediatric Clinic, Klinikum Dortmund, Beurhausstrasse 40, D-44137 Dortmund, Germany; 3 University Hospital of Pediatrics and Adolescent Medicine, St. Josef-Hospital, Ruhr-University Bochum, Bochum, Germany

**Keywords:** Eating skills, Neuromotor development, Dysphagia, Feeding disorder

## Abstract

Early infant feeding and swallowing are complex motor processes involving numerous muscles in coordination, e.g. the orofacial muscles as well as the muscles of the pharynx, larynx and esophagus. The newborn’s reflexive drinking develops into the ability to ingest pureed complementary food as infancy progresses. Finally, in the last part of the first year of life, a differentiated eating, chewing and swallowing process develops allowing the voluntary intake of different foods of the family diet. The dietary schedule for the first year of life, which describes the recommended nutrition of infants in Germany, corresponds to these milestones in eating development. Disturbances in gross motor development, sensory processing issues, and organic and behavioral problems are known to interfere with the development of eating skills. Swallowing disorders (dysphagia) in children can have a detrimental effect on food intake and pose a serious risk to growth and development. Their prevention treatment requires a multidisciplinary approach with the aim of enabling the child to eat independently in the long term.

## Introduction

Infancy and early childhood are periods of rapid body growth and intense neurocognitive development (tripling of birth weight and brain weight by the end of the first year of life). Adequate intake of energy, macro- and micronutrients is an important prerequisite for achieving physical growth potential and for good neurocognitive development [1]. On the other hand, early childhood development of eating and swallowing is a sensitive process. For adequate development of eating skills, certain neuromotor developmental steps are required, which are achieved sequentially in temporally defined, sensitive life stages [2].

The development of the foundations for eating skills begins intrauterine and progresses during infancy and the first years of childhood [3]. Oral sensorimotor skills improve as part of general neurodevelopment, which includes the acquisition of muscle control (including posture and tone), cognition and language, as well as psychosocial skills [4]. However, for efficient oral food intake, adequate general health (including lung and gastrointestinal function) is required in addition to adequate eating skills. Dysphagia in children can adversely affect food intake and pose a risk to growth and development [5].

The first part of the article provides a detailed overview of the developmental processes associated with feeding, from intrauterine development, through infant feeding with the introduction of complementary foods, to family feeding and self-feeding of the child. The second part discusses developmental disorders of feeding and swallowing in connection with neuropediatric diseases and provides suggestions for diagnostics and multidisciplinary care.

## Early development of eating skills

Feeding and swallowing are activities that take place in the upper aerodigestive tract and are controlled by specific areas of the brain and cranial nerves. Recent research suggests that the control of these coordinated movements is based on so-called “Central Pattern Generators” (CPGs). CPGs are neuronal networks, usually located in the spinal cord or brain stem, which are able to autonomously coordinate the activity of many muscles into superordinate movement sequences, i.e., without segmental sensory or supraspinal information. These CPG networks do not operate completely autonomously, but are influenced by segmental afferent signals and by information from cortical-subcortical circuits (review at [6]).

### Prenatal swallowing processes

The development of sucking and swallowing already begins in utero. From about the 13th week of gestation, swallowing movements can be observed; from about the 15th week, the embryo is able to suck on its fingers. After the 20th week of gestation, the tongue movements during swallowing become more complex. Intrauterine swallowing is important for regulating the volume and composition of the amniotic fluid and for the maturation of the fetal gastrointestinal tract. It is estimated that the nearly mature fetus swallows about 500 to 1000 ml of amniotic fluid daily [7].

Fetal sucking movements, characterized by distinct forward and backward movements of the tongue, begin around the 18th to 24th week. The frequency of sucking movements is probably changed by taste influences. Sucking movements are often linked to oral stimulation, e.g. sucking on the finger. Active sucking then develops around the 34th-37th week of gestation; in premature babies, oral food intake via drinking becomes possible at this developmental age [8–11].

## Physiology of early swallowing

Newborns and young children show a different pattern of food intake than adults. This is due to different size ratios, different anatomical structures, and different ways of functioning.

### Preoral phase

An awake, hungry newborn usually signals its hunger by crying, often accompanied by smacking and searching movements. The hungry baby turns towards the mother (the caregiver) and begins to search. The oral reflexes and reactions for searching and sucking are activated. If the baby feels a stimulus on its cheek, it turns its head in the direction of the stimulus and opens its mouth (search reflex). When the nipple touches the lips, the baby begins to open and close its jaw until it feels a stimulus on the tongue that triggers sucking movements (sucking reflex).

### Oral preparatory phase

In the oral preparation phase, food and/or liquid are prepared in the oral cavity by suckling or mastications in order to form a bolus. This includes mixing with saliva, chewing and moving the food posteriorly. In the first months of life, the tongue largely fills the oral cavity and therefore can make only limited forward and backward movements when sucking. During sucking, the soft palate touches the base of the tongue. This both prevents the milk from entering the pharynx prematurely and keeps the airway clear for nasal breathing. If the nasal airways are clear, the infant can coordinate sucking and breathing at the same time during this phase [4, 12].

### Oral phase

During the oral phase (oral transit phase), the bolus is moved posteriorly through the oral cavity. The tongue tip is rests against the alveolar ridge and the bolus is positioned behind it without entering the sulci or pharynx. Milk is held on the median lingual groove and carried in a peristaltic movement along the teat or mammilla towards the pharynx. It is then collected between the velum and the tip of the epiglottis in the area of the valleculae. Only when this area is filled up, the swallowing reflex is triggered [13, 14].

### Pharyngeal phase

During the act of swallowing, the velum raises against the posterior pharyngeal wall, closing off the nasopharynx to prevent nasal regurgitation of milk. The vocal folds and the pouch folds close; respiration is interrupted. The lowered epiglottis directs the bolus laterally into the piriform sinus. The upper esophageal sphincter is relaxed and passively opened by the superior anterior movement of the larynx [14, 15]. The bolus is delivered further into the esophagus through the opened esophageal sphincter by pharyngeal contraction and by the hypopharyngeal suction pumping thrust.

### Esophageal phase

After entering the esophagus, the upper sphincter closes again and the airways are opened. Through peristaltic movements, the food bolus passes through the esophagus and the lower esophageal sphincter into the stomach.

In general, the preparatory and oral phases of deglutition become increasingly voluntary following birth, whereas the pharyngeal and esophageal phases remain involuntary.

## 4. Postnatal development of eating skills and forms of eating

### Milk feeding of the newborn and young infant

With birth, intrauterine drinking behavior must be modified, as the newborn must now coordinate swallowing movements with breathing. Swallowing coordination is one of the most complex neuromotor programs of the newborn. After the 36th week of gestation at the latest, the maturation of the orofacial functions is completed to such an extent that the healthy newborn can take in liquid orally and swallow.

However, the maturation of the orofacial system is not yet complete at birth. Gradually, through learning and experience, neurological maturation, sensory integration and desensitization of the gag reflex, an increasingly consciously applicable motor function develops, which then also enables the swallowing of food of different composition and texture [16].

### Complementary feeding phase

For nutritional reasons, the majority of exclusively breastfed infants require complementary foods from the 5th-7th month of age; the main nutritional arguments are the high iron requirement in the second half of infancy when prenatal iron stores are depleted and the high growth requirement [17].

**Fig. 1:**
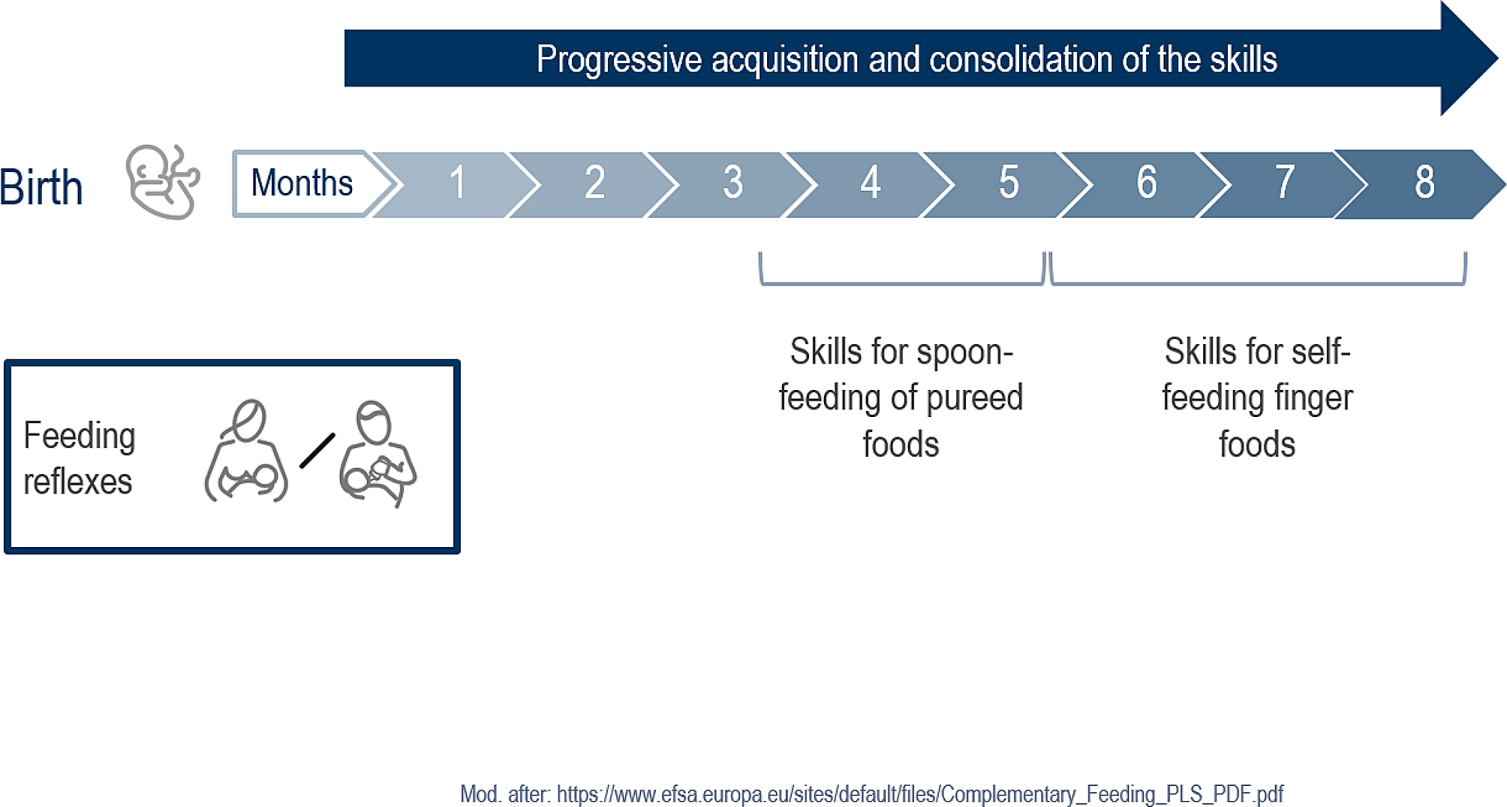
Postnatal development of eating skills

The achievement of several psychomotor developmental steps (‘milestones’) is required for the oral-motor readiness of infants to cope with the transition from a liquid to a pureed diet and later to solid food (Fig. 1). From the age of about 3–4 months, initial suctioning with peristaltic tongue movements decreases and is replaced by suctioning with additional upward and downward movements of the tongue [18, 19].

For the intake of pureed food by spoon feeding, the infant has to move the upper lip downwards to wipe the food off the spoon with his lips (instead of sucking it off the spoon). The mashed food is then picked up on the surface of the tongue, lifted, pressed against the hard palate and then carried to the back of the mouth where the swallowing reflex is triggered. This is a complex movement in which the oral structures must move independently of each other, unlike in pure sucking [20, 21]. A prerequisite for the emergence of these skills is that the child has acquired oral stability to control the jaw, tongue and lips. This develops in parallel with head and trunk stability and control [18, 19].

Sufficient development to spoon feed pureed foods is therefore also demonstrated by the development of gross motor skills in the first year of life, such as adequate head control, improved jaw mobility and the ability to sit or sit with assistive devices [22]. In addition, the protective reflexes present at birth that coordinate sucking, swallowing and breathing and thus reduce the risk of aspiration and choking (so-called extrusion reflexes) must diminish and disappear in favor of freer mobility. Lip, tongue and jaw movements must therefore be sufficiently developed in terms of fine-motor skills for the complex swallowing process [23].

Since taste preferences also continue to develop during the first year of life, infancy may represent a sensitive window for the development of food preferences, which may influence the individual’s long-term potential to establish healthy eating pat- terns; e.g., repeated exposure at an early age may increase the later acceptance of healthy foods [24].

### Introduction of family food

**Fig. 2:**
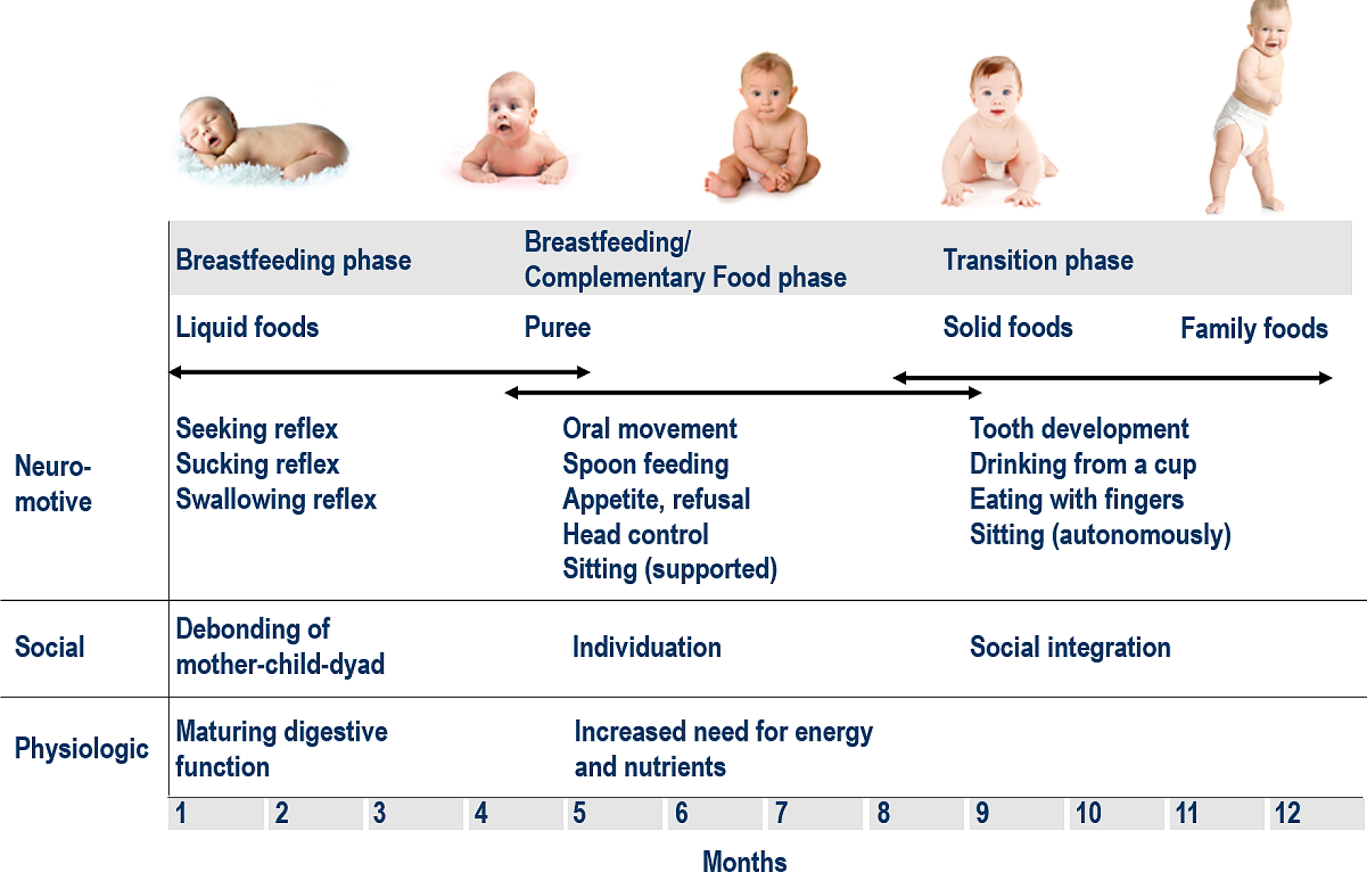
Early childhood development and nutrition [after 28]

As psychomotor development continues in the second half of infancy, the infant’s opportunities for movement continue to increase (Fig. 2). Finally, independent access to food becomes possible (finger food), which is appropriately provided [25]. For the developmental readiness for ‘eating by oneself’ with finger food, the motor skills and coordination of the arm and hand/fingers must be sufficiently developed in addition to the gross motor skills for sitting without support [26]. Towards the end of the first year of life, infants acquire further skills to accept thicker and chunkier food with the spoon. Then a phase of greater independence begins, which is evident in finger feeding of easily soluble solid food. They gradually become more precise in picking up small pieces of food (or other objects) and can form a pincer grip with thumb and index finger, which can be expected at about 10 to 12 months.

In the second half of infancy, chewing movements and first attempts at biting also begin, so that there is increasing acceptance of other consistencies of food. The eruption of the lower incisors also begins at this time; at 12 months, all four incisors are usually visible [27].

Thus, the smooth transition from breastfeeding to self-feeding is developmentally predetermined [28]. The hypothesis of so-called baby led weaning states in its purest form that the infant skips the puree phase and goes directly from breastfeeding to self-feeding of solid food components [29]. However, this would mean that breast milk is still sufficient to provide most of energy and nutrient needed until about the end of the first year of life. Given the developmental milestones outlined above, such a hypothesis is still to be proven and it is unsurprising that there are insufficient data from randomised controlled trials that convincingly prove the safety of baby led weaning in its pure form (the early transition to small solid food components also involves the risk of aspiration).

### The dietary schedule for the first year of life

**Fig. 3:**
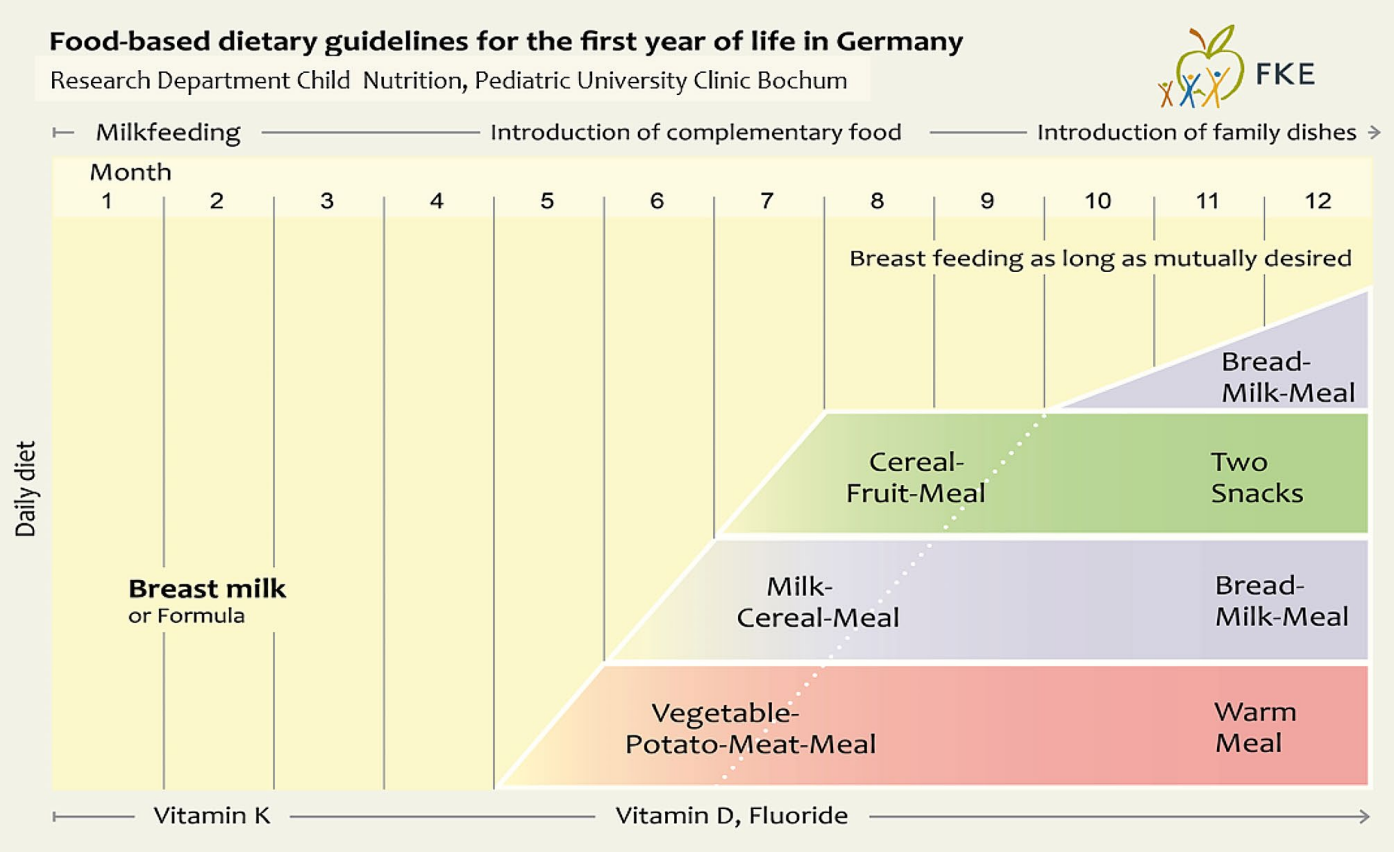
The feeding schedule for the first year of life

The dietary schedule for the first year of life (Fig. 3) comprehensively describes the nutritional development in infancy, taking into account nutrient requirements (inclusive supplementation with Vitamins K, D, and Fluoride), neuromotor development and common foods in Germany.

For reasons of nutritional and developmental physiology, a distinction is made between 3 stages that merge seamlessly into one another:

- Exclusive milk feeding in the first 4–6 months, with breastfeeding as standard,
- Introduction of complementary food from the 5th-7th month with continued partial breastfeeding,
- Introduction of family food towards the end of the first year of life.

For nutritional reasons, complementary feeding starts with an iron-rich meal, which in Germany traditionally contains meat because of its high bioavailability of iron. There is no evidence for the specific exclusion of individual foods (e.g. eggs) in complementary foods to prevent allergies. The high protein intake with the Scheme is caused by the need for nutrient-dense protein-rich foods.

### Eating skills and nutrition after infancy

Between the ages of about two and three years (with the completion of the development of the milk dentition), the so-called infantile swallowing pattern changes to the so-called somatic swallowing pattern. The infantile swallowing pattern is characterized by the tip of the tongue making contact with the anterior teeth on the hard palate; during swallowing, the tongue lies between the rows of teeth and remains inside the mouth without contact with the anterior teeth [30]. The recommendations of the ‘Optimized Mixed Diet’ [31] allow a smooth transition from the infant diet to the family diet, they describe the choice of food and the amounts eaten at the family meals, as in the rest of childhood and adolescence.

## Inter-individual variability in development

**Fig. 4:**
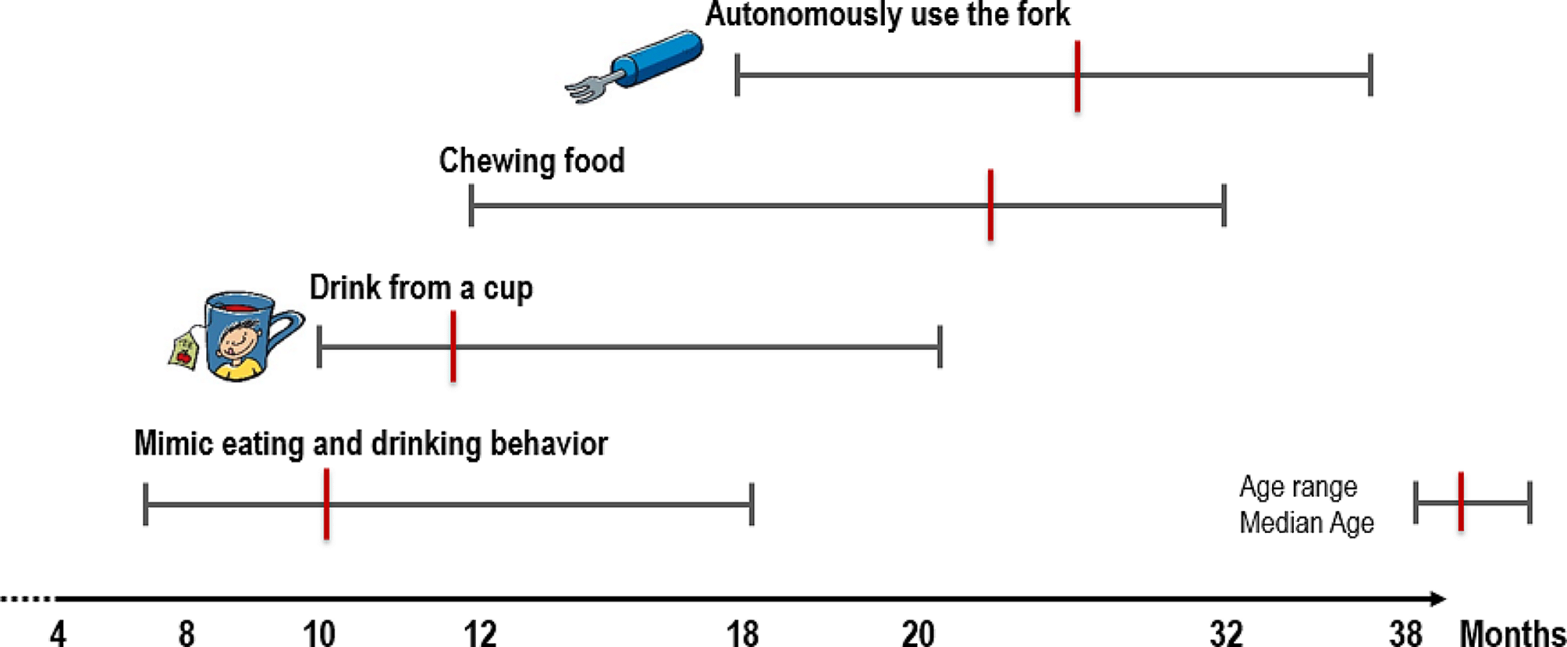
Development of eating skills into toddlerhood (modified after [32])

The age at which infants reach different developmental milestones varies considerably, probably reflecting the infant’s innate developmental trajectory combined with the opportunities and experiences provided by the caregiver (a wide variability is the norm). In addition, feeding skills are acquired and consolidated over time, so that the amount of food an infant eats at the beginning of complementary feeding is small and only increases over time as feeding skills and habituation over repeated experiences increase (Fig. 4). Depending on individual developmental differences, some children eat purees as early as four months, most at five to six months, and some as late as seven to eight months of age [32].

This great interindividual variability in psychomotor development speaks in favor of the time window provided in the Dietary Schedule and confirmed by international pediatric expert groups, rather than a fixed time for the introduction of complementary foods [33, 34]. Autonomous eating develops within a similar range. For instance, some children begin to put food such as bread in their mouths as early as 5 to 6 months of age, but some not until they are 1 year old.

## Disorders in the acquisition of eating skills / dysphagia

Children who “miss” the sensitive phases of learning to eat (e.g., tube feeders) or who have sensory and/or mental development disorders often have difficulty learning to eat and drink.

### Swallowing disorder - dysphagia

Dysphagia is any disorder of the swallowing process that results in an impairment of the safety, efficiency or adequacy of food intake. Importantly, dysphagia is an eating skill-based symptom. This distinguishes it from behavioral eating problems, which can occur even in children who have adequate eating skills. Dysphagia can occur in many patients, such as children with acquired brain injuries or other neuromuscular disorders, craniofacial or respiratory malformations, and children with respiratory, cardiac or gastrointestinal diseases (Table 1). Children with dysphagia may present with several variants of dysphagia affecting one or all phases of the eating process [4, 35]. Micronutrient supplements should be provided orally or enterally (if this can be done safely and effectively).

### Incidence of swallowing disorders

Eating/feeding and swallowing disorders are common; up to 25% of otherwise healthy infants/toddlers may be affected by (usually transient) feeding problems. Many neuromuscular disorders in children (typical leading symptoms are muscle hypotonia, hyporeflexia and weakness) are associated with feeding and swallowing problems, which can lead to a variety of signs and symptoms. It is estimated that about 90% of severe, persistent swallowing disorders in children are associated with a neuromuscular developmental deficit. Common causes of dysphagia are early childhood brain damage with cerebral palsies, neurological diseases, inflammation of the upper alimentary tract such as esophagitis (also reflux-associated, eosinophil-associated) and malformations of the upper alimentary tract. Pronounced feeding problems, especially those that persist into the third year of life, are an important indication of the risk of general developmental delay [36–39].

In children with syndromes, feeding/eating and swallowing disorders are present in more than 50% of cases; suspected causes include prenatal developmental and maturational disorders in various brain areas such as the pons and medulla oblongata. Various dysmorphic syndromes associated with craniofacial malformations are associated with difficult food intake, especially in cases of cleft lip and/or palate [40].

**Table 1:** Possible causes of neurogenic dysphagia (compiled according to [4])

Prenatal	Perinatal	Postnatal
- Syndromes (due to numerical chromosomal aberrations, mutations) -Neuromuscular disorders -Craniofacial malformations -Malformations of brain and spinal cord -Intrauterine intoxications (alcohol, drugs) -Intrauterine infections (e.g. rubella, toxoplasmosis, cytomegaly)	-Hypoxic-ischemic brain injury -Preterm birth	-Infections (meningitis, encephalitis, poliomyelitis) -Traumatic CNS injuries -Tumors -Degenerative CNS diseases -Neuromuscular disorders -Metabolic encephalopathies

### Management

The causes of dysphagia in children often differ from those seen in adult patients. Diagnosis and management of dysphagia must be tailored to the clinical characteristics of the individual patient [26, 41, 42].

Diagnosis requires a detailed history with precise questions about eating behavior, a detailed clinical status and observation of eating; an ENT examination and an endoscopic swallowing examination may also be required. Examinations such as esophago-gastro-duodenoscopy, esophageal pH-metry or manometry are further measures to assess the condition and function of the upper alimentary canal. In addition, the child’s speech development and social behavior must also be included in the overall assessment. Parent reported indicators describing difficulties with eating and drinking are useful to detect children with dysphagia and to monitor progress during therapy [43].

The treatment of eating/feeding and swallowing disorders requires a multidisciplinary approach, with the aim of enabling the child to have autonomy in eating for a longer or long-term period of time [40]. For example, therapeutic interventions for children with swallowing disorders in the oral phase aim to improve the sensory and motor skills needed to drink and eat. For children with swallowing disorders affecting the pharyngeal phase, therapy generally involves modifying the child’s swallowing strategy or changing the food bolus [5, 36]. Dysphagia management should also ensure that food is nutritious and easy to swallow, for example via additional liquid or even texture modification [44].

## Conclusion for practice

For an adequate development of eating skills, certain neuromotor developmental steps are required, which are achieved successively in temporally circumscribed, sensitive stages of life. Feeding and food intake are times of intensive contact between the infant or toddler and the caregiver. The milestones for the development of eating skills are also an expression of increasing independence and early childhood individuation.

During early years, a child’s relationship with food is crucial for his or her health and development. Problems with breastfeeding may already be an indication of (impending) malnutrition [45]. In newborns and young infants, feeding is supported by many involuntary reflex processes; breastfeeding is the natural and ideal feeding for young infants. The transition to complementary feeding is an important developmental step. Increasing oral motor skills are important for this; an upright sitting posture should be supported during feeding. For the developmental readiness for ‘finger food’ and eating by oneself, the motor skills and coordination of the arm and hand/fingers must be sufficiently developed in addition to the gross motor skills for sitting without support. In the second year of life, the toddlers’ eating skills continue to improve so that they can participate in family meals and chew and swallow very different foods of the mixed diet. In general, it can be assumed that achieving the various stages of eating skills in the first years of life is important for the development of later eating habits and therefore may also be important for later nutrition-related diseases [46].

Swallowing disorders (dysphagia) in children can adversely affect food intake and pose a risk to growth and development. Their treatment requires a multidisciplinary approach. The primary goals in the rehabilitation of pediatric feeding and swallowing disorders are to support growth, nutrition and hydration, develop feeding activities and ensure safe swallowing to prevent choking and aspiration pneumonia.

## Data Availability

Not applicable.
